# Ecology and Geography of Transmission of Two Bat-Borne Rabies Lineages in Chile

**DOI:** 10.1371/journal.pntd.0002577

**Published:** 2013-12-12

**Authors:** Luis E. Escobar, A. Townsend Peterson, Myriam Favi, Verónica Yung, Daniel J. Pons, Gonzalo Medina-Vogel

**Affiliations:** 1 Facultad de Ecología y Recursos Naturales, Universidad Andres Bello, Santiago, Chile; 2 Biodiversity Institute, University of Kansas, Lawrence, Kansas, United States of America; 3 Sección Rabia, Subdepartamento Virología, Instituto de Salud Pública de Chile, Ñuñoa, Santiago, Chile; 4 Departamento de Matemática, Universidad Andres Bello, Santiago, Chile; The Global Alliance for Rabies Control, United States of America

## Abstract

Rabies was known to humans as a disease thousands of years ago. In America, insectivorous bats are natural reservoirs of rabies virus. The bat species *Tadarida brasiliensis* and *Lasiurus cinereus*, with their respective, host-specific rabies virus variants AgV4 and AgV6, are the principal rabies reservoirs in Chile. However, little is known about the roles of bat species in the ecology and geographic distribution of the virus. This contribution aims to address a series of questions regarding the ecology of rabies transmission in Chile. Analyzing records from 1985–2011 at the Instituto de Salud Pública de Chile (ISP) and using ecological niche modeling, we address these questions to help in understanding rabies-bat ecological dynamics in South America. We found ecological niche identity between both hosts and both viral variants, indicating that niches of all actors in the system are undifferentiated, although the viruses do not necessarily occupy the full geographic distributions of their hosts. Bat species and rabies viruses share similar niches, and our models had significant predictive power even across unsampled regions; results thus suggest that outbreaks may occur under consistent, stable, and predictable circumstances.

## Author Summary

The situation of rabies in America has been changing: rabies in dogs has decreased considerably, but bats are increasingly documented as natural reservoirs of other rabies variants. A significant gap exists in understanding of bat-borne rabies in Latin America. We identified bat species known to be connected with enzootic rabies with different antigenic variants in Chile, and compiled large-scale data sets by which to test for ecological niche differences among virus lineages and bat hosts. Our results begin to characterize important ecological factors affecting rabies distribution; modeling rabies in Chile allows comparisons across different latitudes and diverse landscapes. We found that rabies virus strains are found in similar environments, regardless of the bat host involved. This research improves understanding of bat-borne rabies dynamics, and important step towards preventing and controlling this and other emergent diseases linked to bats.

## Introduction

Rabies was known to humans as a disease as of about ∼4000 years ago [1]. Although important advances have been made in immunization and diagnosis, rabies is still considered a neglected disease [2]. Rabies is a zoonosis: indeed, although all mammals studied to date are susceptible to infection, major reservoirs that maintain and transmit the virus in the long term are limited to Carnivora and Chiroptera [2]. Rabies virus (RABV) is a neurotropic RNA virus (family *Rhabdoviridae*, genus *Lyssavirus*), including at least 14 species [3]. In the Americas, with generally good control of rabid canines, bats are the main reservoirs of RABV [4]. Rabies transmission from non-hematophagous bats (mainly insectivores) to humans is considered an increasing risk in urban and economically developed areas of Latin America [5], while dog rabies has decreased dramatically in frequency, now occurring only in specific areas of Latin America [6], [7].

Viral “strains” are defined as virus populations maintained by a particular reservoir host in a defined geographic region that can be distinguished from other strains based on molecular and antigenic characteristics [8]. RABV lineages generally show specificity to particular bat hosts [9]–[11]. Antigenic typing depends on use of monoclonal antibodies; their power depends on numbers of monoclonal antibodies that bind consistently to antigenic sites that are conserved in a viral strain [8], [12]. Antigenic characterization is used widely in rabies surveillance in Latin America [9], showing differences among viruses in different host species and geographic locations [13]. *Tadarida brasiliensis*, an important reservoir of rabies in urban areas, maintains antigenic variant AgV9 in North America, but AgV4 in South America [14]. *Lasiurus cinereus* differs, carrying AgV6 across its entire geographic distribution [15]. Viral specificity to these two host species has been confirmed with molecular analyses [9], [10], [13]. These bat species presently constitute the principal rabies reservoirs in Chile [16], [17], but little is known about roles of different hosts in their ecology and distribution. *T. brasiliensis* inhabits sites with other species, roosting in colonies over long periods; owing to anthropogenic perturbation, this species is that which has seen greatest negative population effects in Chile [18]. In contrast, *L. cinereus* avoids urban areas, roost solitarily, and shows seasonal migrations [19]. Both species have broad geographic distributions across the Americas.

Previous such geographic and environmental analyses of rabies lineages have focused on RABV in terrestrial mammal hosts in North America, and documented that rabies in raccoons (*Procyon lotor*) is associated with low wetlands coverage, low elevation, low-intensity residential land use, and absence of major roads, and that rivers act as natural barriers [20], [21].Several studies have explored features of host-virus relationships of bat-borne rabies, based on molecular genetic analyses [22]–[25]. However, in these key studies, inferences about geographic pattern were made based on points on an empty map, without reference to environmental drives. Hence, landscape- and niche-based approaches could offer a valuable complement to conclusions generated in molecular genetic studies, evaluating effects of environment and landscape on rabies host and virus distributions, but such methods must be explored and validated first.

To test these approaches, we address a series of questions regarding rabies transmission ecology in Chile. (i) Do rabies lineages have coarse-grained ecological “signatures” (i.e., Grinnellian niches) that can be characterized robustly? (ii) Do macro-ecological and macro-geographic linkages exist among viruses and hosts? Finally, (iii) do different bat-borne rabies lineages have distinct ecological signatures? Answering these questions will help to illuminate details of virus-host dynamics in bat rabies transmission cycles in South America.

## Methods

In recent years, several innovations have converged in making possible improved understanding of environmental conditions required by organisms to maintain populations, including rich data streams by which to characterize environments, powerful inferential tools, and increasingly comprehensive conceptual frameworks [26]. These developments allow researchers to characterize relationships between species' occurrences and environmental variables, as an approach to estimating dimensions of species' ecological niches and, by extrapolation, their geographic distributional potential [26]. Via such “ecological niche modeling” approaches, various pathogens, vectors, and reservoirs have been analyzed to understand how environmental conditions relate to disease transmission [27]. Niches seem generally to show relatively slow evolutionary change [28], another element in making these analyses feasible. Hence, in this study, we use ecological niche modeling to assess the degree to which distribution of host and virus lineages are associated consistently and predictably with particular sets of environmental conditions—i.e., that they respond to a consistent and predictable ecological niche.

### Study area

**Figure 1:**
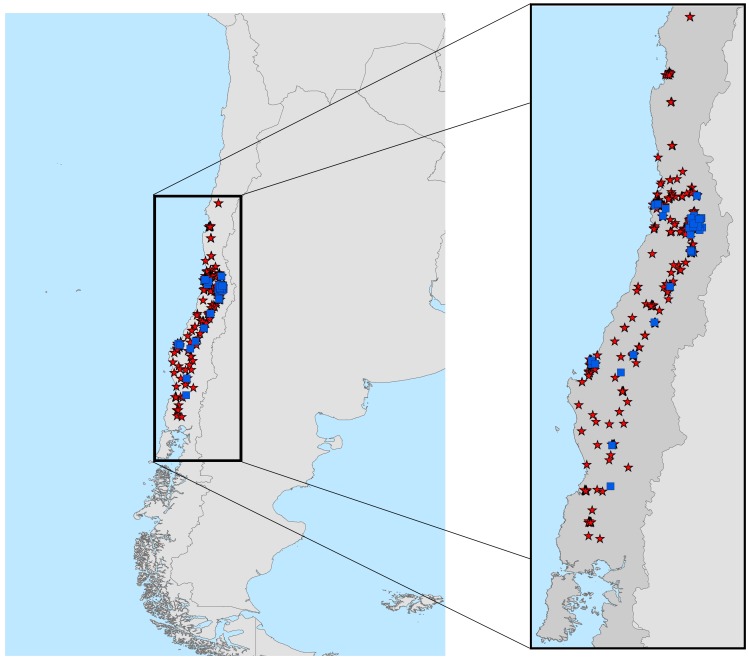
Occurrences in study area. Rabies occurrences across the study area in central Chile: AgV4 (red stars) in *Tadarida brasiliensis*, and AgV6 (blue squares) in *Lasiurus* spp.

Delimitation of the geographic area of analysis is a crucial issue in generating robust niche models, with significant effects on model results [29]. The study area must be established *a priori* based on (1) the dispersal potential of the species involved, (2) the sampling available by which to characterize distributions, and (3) the objectives of the study [29]. We delimited our study area to the area between −28.0° and −43.5°s latitude in Chile, corresponding both to the enzootic area in recent decades [16] and to the area sampled by the Chilean Ministerio de Salud (Ministry of Health; Fig. 1).

### Input data

Another crucial aspect in niche model development is the set of environmental variables used to characterize the environmental space in which the species is distributed [30]. We used information on land-surface reflectance from remote sensing, in light of its high information content, fine spatial resolution, and minimal need for interpolation and inference [31]. Environmental variation can be summarized using multiple seasonal values of the Normalized Difference Vegetation Index (NDVI), which has values correlating strongly with photosynthetic mass and primary productivity [31], [32]. Numerous previous studies have shown the importance of such vegetation indices as indicators of ecological and geographic dimensions [31], including in development of robust ecological niche models [33], [34]. We used NDVI images available as monthly maximum raster data layers for 1992, 1993, and 1995, which correspond to the middle years of the study period, at a spatial resolution of 0.01°×0.01°; to standardize these variables and reduce dimensionality, we generated principal components across all of the monthly data sets using ArcGIS 9.3 (ESRI, Redlands, CA, USA). Principal components analysis used the original NDVI layers to generate 27 new, uncorrelated components: we used the first 10 components in model development (i.e., the initial 10 axes that best characterized the major dimensions of the cloud of points), as they explained 99.99% of overall variance.

To characterize spatial patterns of bat-rabies occurrence across Chile, we only digitized bat surveillance data from the Instituto de Salud Pública de Chile (ISP), for 1985–2011, corresponding to the major enzootic period for bat rabies in Chile (Fig. 1). Host mammal occurrences were obtained from both active and passive surveillance programs, with hosts tested for rabies and identified at ISP. Coordinates of bat occurrences (both species, regardless of rabies status) were derived from geographic centroids of municipalities, as they were submitted by municipal agencies for testing. Further occurrences were obtained through data mediated by the Global Biodiversity Information Facility (GBIF; see Acknowledgments for full list of institutions), with georeferencing derived from original data records.

Virus occurrences were obtained in the form more precise georeferences derived from postal addresses of sites of origin of rabies-positive bats of both species, although the vast majority (78%) came from *Tadarida*. These cases were diagnosed by ISP using direct inmunofluorescence (IFD), to confirm virus presence, and monoclonal antibodies to identify virus variants [35].

### Model calibration

To calibrate niche models, we used a maximum entropy algorithm, considering its predictive power and broad acceptance in the scientific community [36]. The algorithm uses the information theory concept of maximum entropy to optimize estimates of suitability across complex environmental spaces. The maximum entropy approach seeks to estimate the probability of suitability through finding the probability distribution closest to uniform, subject to certain restrictions; in our case, the restrictions are environmental conditions associated with known occurrences of the species in question [37].

In Chile active surveillance is initiated after a positive bat is reported from passive surveillance. ISP samples originated from passive surveillance [16], [17] associated with human settlements, without anything close to uniform geographic coverage. We incorporated sampling bias across the study area in model calibration because spatial and environmental biases in data collection can cause biases in model results [38]. Maxent can use a sampling bias distribution (*σ* in Phillips et al., 2009) to establish areas from which to focus extraction of background data with which to calibrate models [38]. We thus developed a sampling bias surface for *T. brasiliensis* based on all of the passive surveillance data, using overall numbers of samples submitted to ISP per municipality (municipalities with no samples set to no data, and thus excluded from background sampling), regardless of rabies-positive status, on the final raster, we added 1 to all pixels to avoid zero values, according to Maxent requirements. This surface appropriately characterized the sampling that underlies the virus-positive records that drove calibration of the niche models. We calibrated models with and without this bias file to assess the degree to which sampling effort affects results.

We calibrated models using Maxent version 3.3.3.k. Specific options were a bootstrap subsampling with 1000 replicates, random seed, and the median of replicates as output. We converted raw Maxent output to binary maps considering an error rate of *E* = 10% among occurrence points, and thus used the highest threshold that included 90% of training presence points [26], a modification of the least training presence threshold idea [39]. The error rate (*E*) is the proportion of the occurrence data expected to place the species erroneously under inappropriate conditions, as a consequence of incorrect species identifications, errors in georeferencing, and errors in environmental data, among other factors, and is estimated via exploration and error-checking of the occurrence data [40]. We visualized ecological niche models in environmental spaces based on plots of NDVI values in winter and summer from across the study area, comparing this environmental ‘background’ with corresponding values associated with known occurrences of bat species and rabies variant.

### Model evaluation

Niche models must be evaluated to validate their predictive power, before any use or interpretation [26]. We evaluated the predictive ability of models for *T. brasiliensis*; however, sample sizes for *L. cinereus* were too small and too clumped spatially to permit detailed evaluations. Two different spatial subsetting schemes were explored, taking advantage of the roughly linear shape of Chile. First, we subset data latitudinally by quintiles of frequency, dividing occurrences into five subsets, and using subsets 1, 3, and 5 for model calibration and subsets 2 and 4 for evaluation [26]. Second, we divided the study area into five equal-width latitudinal bands, again using subsets 1, 3, and 5 for model calibration and 2 and 4 for evaluation. In the first scheme, subsets had equal sample sizes, whereas in the second scheme, subsets had similar areal dimensions (Fig. S1 for supporting information).

For evaluating models, we avoided traditional receiver operating characteristic (ROC) area under the curve (AUC) approaches, considering that AUC tests require presence and absence data for proper implementation [41], and in light of recent critiques [40], [41]. Rather, models were first evaluated using areas and points predicted as suitable and unsuitable after thresholding (based on *E* = 10%) using a cumulative binomial probability distribution [26]. Second, models (without thresholding) were evaluated using partial ROC approaches [42], [43], evaluating the predictive ability of niche models considering only omission errors and proportional areas predicted as suitable, and only over a range of omission errors deemed acceptable in light of error characteristics of the input data (here again we used *E* = 10%, and thus allowed up to 10% omission in our partial ROC calculations). In partial ROC, the area under the observed line of model performance is related to the area under the line of random expectations, and a ratio is calculated. Bootstrap manipulations (1000 total), in which 50% of evaluation data are resampled with replacement and AUC ratios recalculated, are used to test the hypothesis that model performance is better than random expectations. When ≥95% of bootstrap-replicate AUC ratios were >1, we rejected the null hypothesis of performance no better than random expectations [42]. Partial ROC software is available for free download in http://kuscholarworks.ku.edu/dspace/handle/1808/10059

### Niche model comparisons

Finally, to compare niche models between virus strains and bat species, we used niche identity tests to determine whether two niche models are indistinguishable from one other [44]. Identity tests have the advantage of restricting comparisons to the same set of points, a feature that is particularly relevant for our occurrence data, which did not come randomly from across the entire landscape. We calculated observed Hellinger's modified (*I*) and Schoener's (*D*) distances between niche models (thresholded using minimum training presence approaches), and compared them to a null distribution of comparable distances derived from 1000 replicate random subdivisions of the overall pool of occurrence data between the two species, maintaining observed sample sizes. We used ENMTools (version 1.3; http://enmtools.com) for these comparisons [45]. We evaluated whether niche characteristics were identical between rabies lineages (AgV6 *versus* AgV4), between the host species and associated viruses, and between the two host species. In all comparisons, our critical value was the 5^th^ percentile of similarity (i.e., low end), as we were seeking evidence of niche differentiation [45].

## Results

### Ecological signatures

In all, 26,323 bat samples from active and passive surveillance were submitted to ISP during 1985–2011, a data set that was captured digitally as part of this study. However, many records corresponded to the same county centroids, such that sample sizes were nowhere near the number of samples: in all, to model hosts, we found 70 unique occurrences for *L. cinereus* (9% from GBIF; 91% from ISP) and 238 for *T. brasiliensis* (3% from GBIF and 97% from ISP). For rabies samples, we obtained 910 unique coordinates for rabies AgV4 (bat rabies-positive associated with *T. brasiliensis*) and 52 for rabies AgV6 (associated with *Lasiurus* spp.; Fig. 1); sample sizes are larger in this case because georeferencing was to street addresses, rather than just to county centroid.

**Figure 2:**
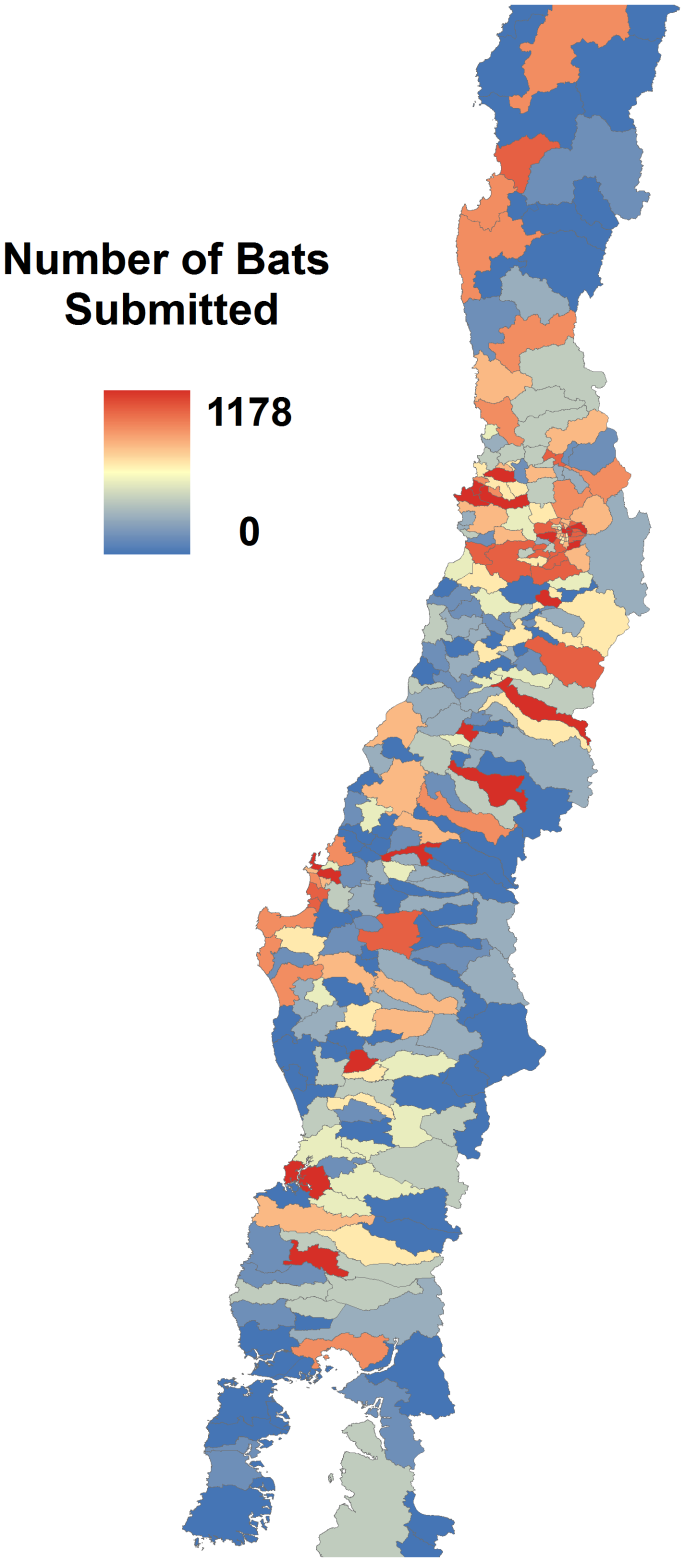
Bats submitted by municipality since 1985 to 2011. Sampling intensity of *Tadarida brasiliensis* bats by municipality, used as the sampling bias grid in Maxent analyses.

**Figure 3:**
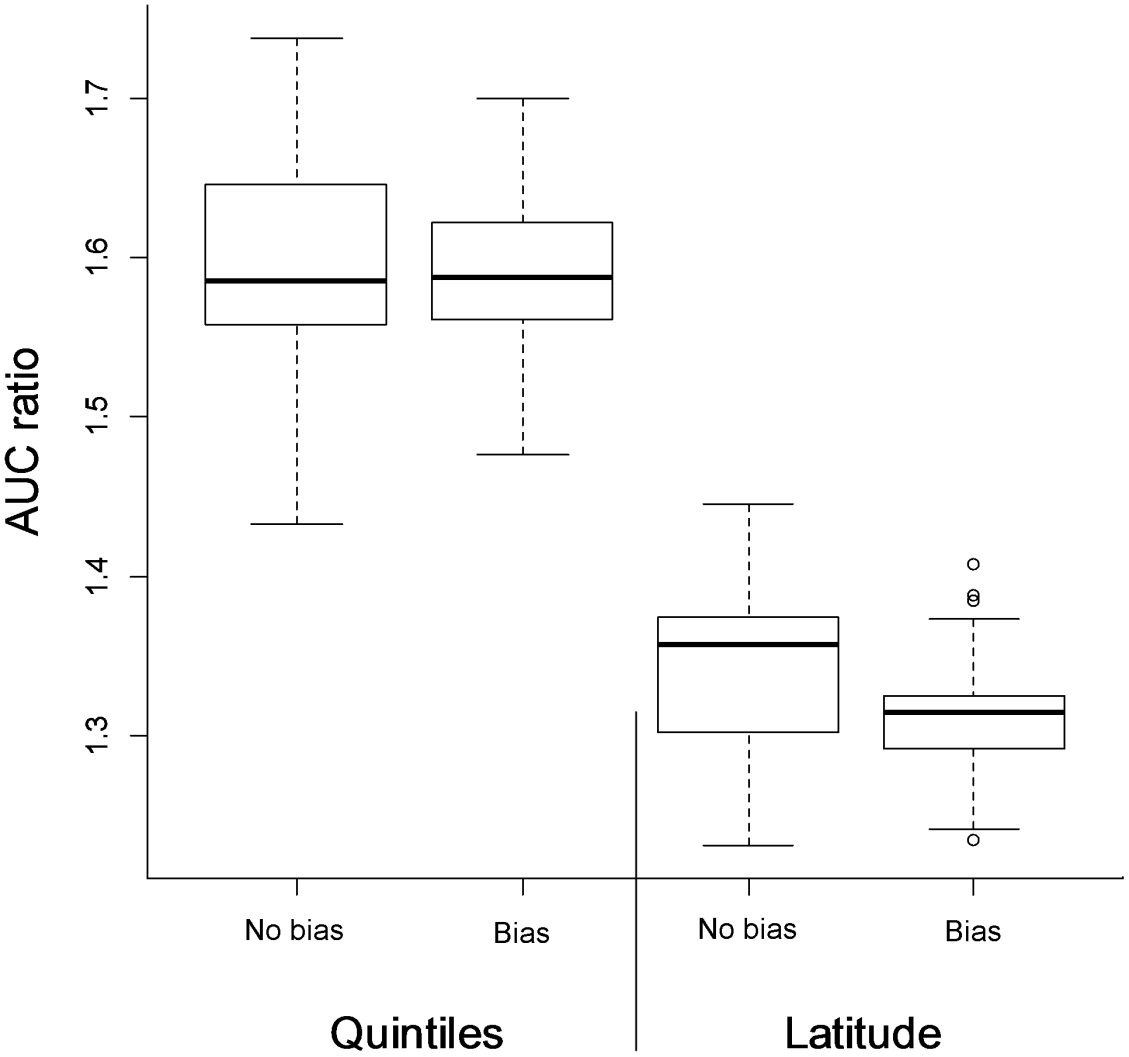
Partial ROC results in model evaluations. Evaluation of niche models for AgV4 rabies in central Chile, using different calibration areas (quintiles and latitude subsetting), and comparing models developed with (Bias) and without (No bias) consideration of sampling bias.

**Figure 4:**
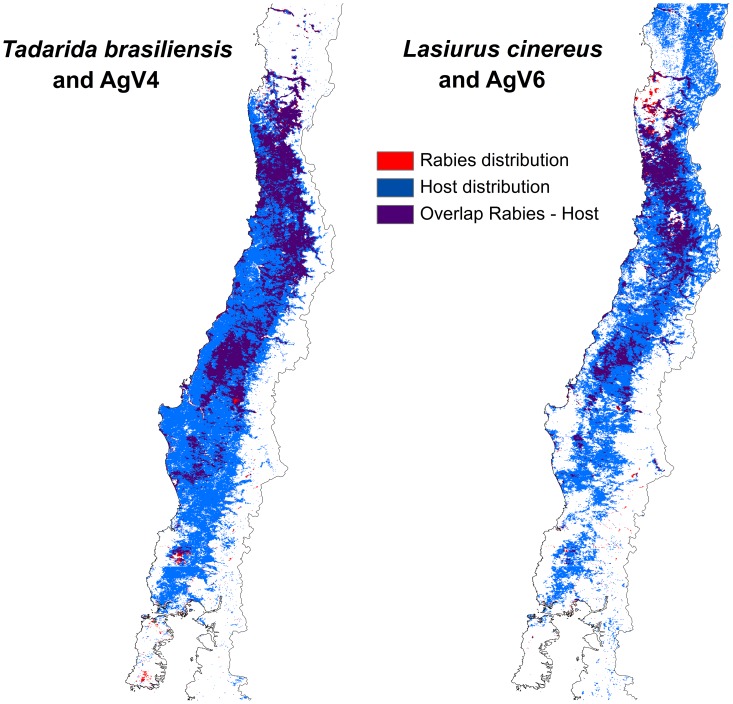
Distribution map of rabies and its hosts. Maps of potential distribution of hosts (blue), rabies strains (red), and overlap of host-rabies distribution (purple).

Sampling intensity for *T. brasiliensis* varied 0–1178 samples submitted per municipality (Fig. 2), while that for *L. cinereus* varied 0–164; with only 64 of the 301 counties in the study area submitting *L. cinereus* samples. Niche models, whether considering sampling bias or not, all performed significantly better than random expectations, with partial ROC AUC ratios associated with our niche models were >1 (Fig. 3). However, considering that models controlling for sampling bias generated predictions with smaller suitable areas, we prefer to use these models in further steps. For example, quintile subsetting considering sampling bias had less area predicted (35.2% of the study area) than comparable models without considering sampling bias (38.0% of the study area). Bias control also resulted in lower variance in AUC ratios in the partial ROC analyses (Fig. 3). With this general confirmation of predictive power, we proceeded to build ecological niche models for each species (Fig. 4) for interpretation.

### Ecological linkages and differences between viruses and hosts

**Figure 5:**
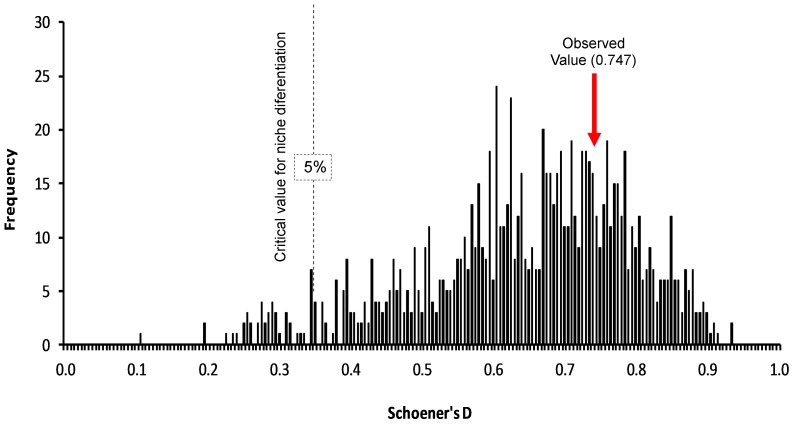
Histogram of *D* similarity values among random replicates in testing niche identity between rabies AgV4 and AgV6. Note that the observed value is well above the critical value in testing our null hypothesis of niche identity.

None of the six identity tests comparing niches between the two host species, between each host species and its associated virus linage, and between the two virus lineages, was able to reject the null hypothesis of niche “identity” (Table 1). Figure 5 shows the latter comparison graphically: observed similarity fell well above the critical value in all comparisons. In sum, at least across central Chile, the two bat species and their associated viruses share very similar ecological niches, at least in the coarse-grained environmental dimensions explored in this study.

**Table 1:** Results of niche identity tests assessing similarity between occurrences of *Tadarida brasiliensis* and *Lasiurus cinereus* and rabies strains AgV4 and AgV6.

	*I*				*D*			
	Obs	5%	95%	P value	Obs	5%	95%	P value
**V6/V4**	0.862	0.588	0.909	P≥0.05	0.747	0.346	0.831	P≥0.05
**Lc/V6**	0.891	0.815	0.934	P≥0.05	0.834	0.669	0.908	P≥0.05
**Tb/V4**	0.892	0.839	0.974	P≥0.05	0.888	0.707	0.967	P≥0.05
**Tb/Lc**	0.836	0.827	0.938	P≥0.05	0.757	0.709	0.925	P≥0.05

**Figure 6:**
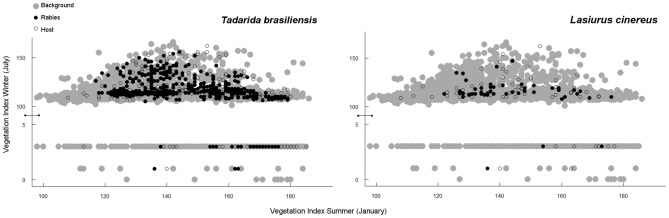
Host and virus distributions in environmental spaces. Distribution of hosts (unfilled points) and corresponding rabies variants (black points) across the environments available in our study area (background; gray points), for *Tadarida brasiliensis* (left) and *Lasiurus cinereus* (right). Environmental variation was visualized as bivariate comparison of NDVI values for January (summer) and July (winter) in the southern hemisphere.

The two bat species had broad distributions in environmental space (Fig. 6). Rabies infections were found across the great bulk of the environmental distribution of each of the hosts. However, both hosts appear to avoid areas presenting extremely low NDVI values in summer and winter, corresponding to the high Andes regions.

## Discussion

In Chile, rabies has been reported as far back as 1879 [46]. All data have been centralized in the Sección de Rabia, Instituto de Salud Pública, since 1929 [17]. Via effective monitoring, mass dog vaccination, elimination of biting stray dogs, improvement of diagnosis quality, and post-exposure vaccination in humans, urban canine rabies was eradicated as of about 1990 [47], [48]. However, over the same period, the zoonotic cycle, wherein the main reservoirs are bats, has been increasing in importance [16]. Hence, in Chile, reports suggest rabies in a process of re-emergence in the wildlife cycle [16], [17], [49].

Our large-scale data set, broad latitudinal gradient, and dramatic diversity of landscapes and biomes across the study area allowed a robust test and validation in the use of niche modeling in understanding the spatial epidemiology of bat-related rabies, as required when modeling diseases [50]. Answering our first question, it was possible to characterize ecological niches of rabies viruses and their hosts consistently and with good predictive power. In the broadest sense, niche models for the two bat species confirmed the obvious: the high Andes Mountains in the east and the Pacific Ocean in the west are natural barriers [18], while the Atacama Desert to the north and cold regions in the south delimitated our study region naturally [29]. With this definition of relevant areas, we derived clear predictions of the geographic distribution of both bat species (Fig. 4), wherein *T. brasiliensis* may be somewhat more limited in its use of cold and high zones in the Andes and the northern deserts than *L. cinereus* (Fig. 4). The broad suitable areas for both species corroborate the ecological plasticity known in bats [51] and migratory behavior reported in the northern hemisphere for both *T. brasiliensis* and *L. cinereus*.

Niche models provided a first view of rabies distributions in geographic and environmental spaces [27]. Our ecological niche models for rabies lineages using fine-resolution satellite imagery identified putative potential areas of rabies distribution, albeit under stable characterizations of environments averaged across several years of conditions; clearly, more dynamic characterizations of rabies distributions merit future evaluation. Although we assembled large data sets that are reasonably comprehensive for Chile, we hasten to point out potential gaps and failings in our data and analysis. A first such caution is that of the uneven spatial and environmental distribution of rabies in Chile: although samples were submitted from across the county, rabies locations were mainly from passive surveillance, producing three clusters of rabies cases in the main cities of central Chile (Santiago, Valparaiso, Concepción; Fig. 1), biases that we took into account in our analyses. Using the bias file helped to reduce variance in model performance, allowing clearer discrimination of performance between models (Fig. 3). We used sampling bias summaries for *T. brasiliensis* to consider the availability, quantity, and quality of data available for this species; for *Lasiurus*, parallel data were not available in sufficient quantity, reflecting the relative rarity of sample submissions for that species. Incorporating information on sampling intensity in niche modeling for public health applications is an issue that merits further exploration, particularly considering that the more biased the data are, the more benefit that derives from use of sampling bias surfaces. Our improvements in model performance with bias surfaces were analogous to previous results in biodiversity studies [38]. As result, our models provide at least a preliminary assessment of risk in several areas that currently represent gaps in surveillance [52].

Ecological niche models have seen detailed performance testing in challenges centered on estimating niches and predicting species' distributions, showing impressive success even in spite of spatial sampling biases (e.g., sampling along roads) [53], [54]. Problems arise when sampling is biased with respect to environments, however, since models based on such sampling will be effectively blinded to potential for occurrence in unsampled environments [53], [55]. An additional source of potential problems is the precision of georeferencing that was possible for these data, considering that reports of disease occurrence may simply provide the patient's address, but not necessarily the site of infection, which is more relevant in spatial epidemiology [56]. In this study, such problems introduce a basement level of spatial accuracy in model predictions, such that finest-resolution phenomena may not be “visible” in results.

In relation to our second question, it is important to note that, although viruses and hosts share ecological niche characteristics, the virus does not necessarily occupy the full host distribution (Fig. 4); the geographic bias, however, at least within our study area, appears to be without consistent environmental correlates. Our methodology corroborates the rabies-bat relationship that has heretofore gone untested at landscape scales, and our results suggest that niche modeling offers a useful tool for mapping disease occurrences and potential for occurrence in public health [27]. With respect to our third question, niche identity tests between hosts and viral variants indicated that niches of all actors in the Chilean bat-rabies system are similar in environmental requirements; that is, we were unable to reject the null hypothesis that niche models of host species are not different from niches of associated virus strains, and indeed that the two host species and the two virus strains do not differ from one another either.

Currently, little is known about the ecology and transmission of rabies virus among bats, but phylogenetic evidence gives strong indications of host specificity [9], [13]. In this sense, not only do rabies virus variants appear to track the ecology of their respective hosts, but also the pairs of viruses and hosts do not differ from one another. A recent report offers some corroboration of this assumption via molecular analysis: a rabies strain specific to *Lasiurus* spp. bats was found in *T. brasiliensis* in Chile [13], which indicates cross-species spillover transmission of virus lineages in taxonomically distant bat species under natural conditions. These results support the idea that rabies viruses may infect hosts without environmental bias (see [44], for parallel results).

Restating, the bat species and rabies lineages evaluated appear to share very similar portions of environmental space, even if this result is not manifested as complete overlap in geographic space (Fig. 4), perhaps because different geographic distributions do not necessarily reflect niche differences [28]. This result allows a view into how rabies host ecology influences virus biology, and suggests that taxonomic differences in hosts or viruses do not necessarily translate into ecological differences. Our results and those of similar studies [51], [57] may help to clarify the ecology of bat rabies lineages in other hosts and geographic regions. Potential distribution maps of hosts and their viruses can be an important tool by which to understand potential transmission areas for rabies, although these approaches remain little explored [51]. Bat-borne rabies has seen some events of cross-species transmission in zoonotic cycles in Chile, with AgV 4 (related to *T. brasiliensis*) found in *Lasiurus* spp. and AgV 6 (related to *Lasiurus* spp.) found in *T. brasiliensis* [10], [13]. Accidental hosts have also been reported in recent years: for instance, mortality of dogs, cats, farm animals, and a human caused by rabies related to *T. brasiliensis* [10], [13]. Via this scenario, control of stray dogs and feral cats as well as vaccination campaigns must be implemented with priority in those areas where host and virus distribution match (Fig. 4).

In conclusion, one should take care to avoid the logical, scale-related error that can be termed the “Beale fallacy.” Beale et al. [58], analyzed distributions of European birds with respect to climate, and concluded that their distributions were not limited by climate. While this conclusion was, to some degree true, it was completely dependent on the particular context of Western Europe and relatively broadly-distributed bird species; a parallel analysis in a different context found abundant climatic determination of ranges [59]. In this sense, our conclusion about no niche difference among our bat species and rabies lineages must be considered as context-dependent [59]: analyses over broader regions may well detect clear and significant differences. Our results show two viral lineages as sharing similar environmental signatures with two bat host species, regardless of antigenic characteristics, known associations, and phylogenetic position. Recent years have seen important advances in molecular dimensions of studies of rabies, but few have explored how regional landscapes affect (or not) distributions and dynamics of rabies in zoonotic cycles [20], [21]. In light of the results reported herein, the spatial epidemiology and ecology of zoonotic bat rabies should see further exploration.

## Supporting Information

Figure S1 Distributions of calibration and evaluation areas, based on latitude (left), and based on quintiles of frequency for model evaluation.
(TIF)
