# Supplementary figures and images for: Ecology and Geography of Transmission of Two Bat-Borne Rabies Lineages in Chile
Source: PLoS Negl Trop Dis. 2013 Dec 12;7(12):e2577. doi: 10.1371/journal.pntd.0002577 (PMC3861194; doi:10.1371/journal.pntd.0002577)

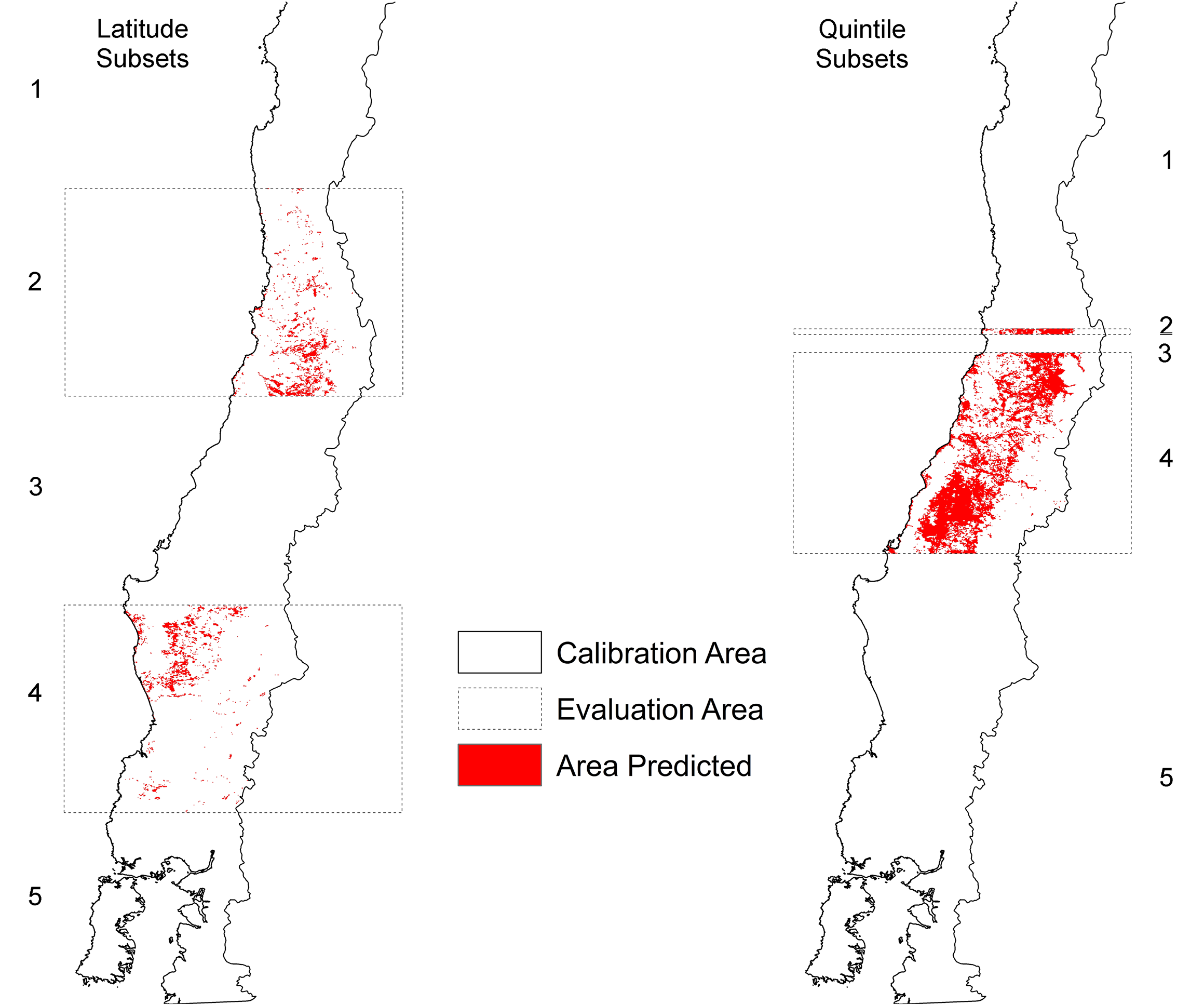

Supplement: Figure S1 — Distributions of calibration and evaluation areas, based on latitude (left), and based on quintiles of frequency for model evaluation. (TIF) [file pntd.0002577.s001.tif]
